# Impaired remyelination in late-onset multiple sclerosis

**DOI:** 10.1007/s00401-025-02868-5

**Published:** 2025-04-01

**Authors:** Lidia Stork, Schirin Stephan, Adriane Kutllovci, Wolfgang Brück, Imke Metz

**Affiliations:** Institute of Neuropathology, University Medical Center Göttingen, Robert-Koch-Str, 40, 37075 Göttingen, Germany

**Keywords:** Late-onset multiple sclerosis, Inflammation, Acute axonal damage, Axonal density, Remyelination, Oligodendrocytes, BCAS1

## Abstract

A reduced regenerative capacity may contribute to faster disease progression and poorer relapse recovery in multiple sclerosis patients with disease onset after the age of 50, a condition known as late-onset multiple sclerosis (LOMS). We hypothesized that lesions in LOMS patients show more pronounced axonal damage, less remyelination and an altered inflammatory composition, and performed a detailed histopathological analysis of MS biopsies in patients with early-stage LOMS. The number of T cells, B cells, plasma cells, microglia/macrophages, different oligodendrocyte populations as well as the axonal density and acute axonal damage were assessed in 31 LOMS and 30 normal-onset MS (NOMS, 20–40 years old) patients. No major differences in the inflammatory infiltrate or axonal damage were found. BCAS1-positive oligodendrocytes indicating early myelinating oligodendrocytes, and mature oligodendrocytes were significantly lower in the normal-appearing white matter of LOMS compared to NOMS patients (*p* = 0.05; *p* = 0.01), with a negative correlation with age (r = − 0.5, *p* = 0.01). In active demyelinating lesions, the number of BCAS1-positive oligodendrocytes did not differ between LOMS and NOMS, but NOMS lesions showed a higher proportion of ramified cells indicating active remyelination. In LOMS, BCAS1-positive oligodendrocytes decreased with increasing lesion age, with the lowest numbers found in inactive demyelinated lesions. In contrast, NOMS patients showed high numbers of BCAS1-positive cells with an activated morphology, even in inactive demyelinated lesions. At the last follow-up, LOMS patients had a significantly higher EDSS score (median 3.5) than NOMS patients (median 3.0, *p* = 0.05). A higher EDSS score correlated with fewer mature and oligodendrocyte precursor cells in active demyelinating lesions (r = − 0.4, *p* = 0.01 and r = − 0.6, *p* = 0.003). These findings suggest a clinically relevant impaired oligodendrocyte differentiation and remyelination in LOMS. Since remyelination is essential for axonal protection, it will be necessary to consider the complex and dynamic tissue environment when researching therapeutics aimed at fostering the differentiation of oligodendrocyte precursor cells into myelinating oligodendrocytes.

**Supplementary Information:**

The online version contains supplementary material available at 10.1007/s00401-025-02868-5.

## Introduction

A patient’s age at the onset of multiple sclerosis (MS) is a significant factor that influences pathological processes associated with MS as well as disease progression. The disease typically manifests between the ages of 20 and 40, which is defined as normal-onset MS (NOMS); when it manifests after age 50, it is classified as late-onset MS (LOMS). The prevalence of LOMS varies considerably across studies, with estimates ranging from 4.6% to 11% of all MS patients [27, 30, 43]. LOMS is associated with a more neurodegenerative disease phenotype. In these patients, a primary progressive disease course is more frequently observed, and the conversion from relapsing–remitting MS (RRMS) to a secondary progressive disease course (SPMS) occurs at a faster rate [16, 26]. In LOMS the disease typically manifests initially with motor or sensory symptoms, with a higher disability score at disease onset [16, 27]. Patients demonstrate a less favorable recovery from relapses and a less robust response to disease-modifying anti-inflammatory therapies [8, 11, 16, 39]. A less inflammatory phenotype might be suggested as MRI shows fewer gadolinium-enhancing lesions, indicative of blood–brain barrier leakage and active inflammation. In addition, lower CSF cell numbers are found in LOMS compared to NOMS [16]. Also, there is generally less response to corticoid therapy [16]. Please refer to the Supplementary Table 1 for a more comprehensive literature analysis comparing the clinical data of LOMS and NOMS patients.

Despite these obvious clinical differences, there are only a few histological studies on LOMS pathology. One neuropathological study on the early disease stage of LOMS demonstrated early active demyelination in 63%, and cortical demyelination in 58% of patients [41]. In a postmortem clinical-pathological study of late MS stages, LOMS pathology was characterized by a lesser extent of overall demyelination, yet a greater proportion of cortical demyelination in comparison to NOMS [17]. Furthermore, LOMS patients showed a lower number of active or chronic active lesions [17].

Neurodegeneration, driven by demyelination and inflammation, is initiated at the onset of the disease. It is hypothesised that acute axonal damage and partial axonal loss act as a trigger for subsequent neurological progression [12, 18, 24]. To our knowledge, investigations of the axonal damage in LOMS have not been performed. However, a reduction in neuronal density was observed in the cingular gyrus and thalamus of LOMS patients [17]. The integrity and functionality of axons may be compromised by the loss of trophic support resulting from demyelination and by inflammatory processes. However, a lower extent of leptomeningeal and perivascular inflammation was observed in LOMS patients in late MS stages [17], which does not explain the more neurodegenerative clinical phenotype observed in LOMS.

Remyelination is an endogenous repair mechanism that is initiated at an early lesion stage, even in the context of active demyelinating lesions [2]. Remyelination serves to protect axons and facilitates clinical recovery after a relapse. More pronounced remyelination is associated with a reduction in clinical disability in patients with MS [3, 4]. However, the extent of remyelination varies considerably between patients and is associated with disease duration, disease course, and patient age [29]. Remyelination is most pronounced in the early stages of the disease and then decreases as the disease progresses [15]. Whether LOMS patients differ from NOMS patients in their ability to remyelinate in early disease stages has not been investigated, although studies have shown that the ageing of glial and immune cells has a profound effect on central nervous system (CNS) function.

It seems reasonable that LOMS patients have histopathological changes associated with the ageing process that contribute to the development and progression of the disease. We hypothesised that the neurodegenerative phenotype of LOMS is characterised by a more pronounced axonal damage and/or axonal loss, as well as less remyelination and an altered inflammation. Therefore, we performed a comprehensive histological study comparing the pathology of early disease stages of LOMS and NOMS patients to exclude the effects of a longer disease duration in older patients. We examined the number of immune cells, the extent of axonal damage, and the diversity of oligodendrocyte populations within MS lesions with different demyelinating activities. The analysis included a detailed examination of early myelinating oligodendrocytes, with particular attention paid to their morphological characteristics. While no clear differences in the immune cell composition and axonal damage were observed in LOMS compared to NOMS patients, the results indicated that age primarily affected oligodendrocytes and their remyelination capacity. We found that the number of mature and early myelinating oligodendrocytes was significantly lower in LOMS patients, and a lower number of oligodendrocytes was associated with a higher level of disability. Morphological analysis also suggested an impaired remyelination in LOMS.

## Materials and methods

### Study cohort

The study was approved by the Ethics Committee of the University Medical Center Goettingen (#19 /09 /10). Formalin-fixed, paraffin-embedded archival tissue from patients with inflammatory demyelinating lesions consistent with MS and diagnosed by brain biopsy was analysed. All included brain biopsies were performed for differential diagnosis in a clinical setting, e.g. to exclude tumor or infectious disease.

Human brain biopsies were taken between 2006 and 2016 at various neurosurgical centers in Germany and sent to the Department of Neuropathology at the University Medical Center Goettingen for a second opinion. A total of 67 patients were included in the study. The initial cohort of LOMS patients consisted of 28 patients with the first disease manifestation at the age of ≥ 50 years. To analyse remyelination in three different demyelinating lesion stages (early and late active demyelinating as well as inactive demyelinated lesions), the cohort was expanded with 6 LOMS patients (total *n* = 34, 4 patients from the initial cohort did not have sufficient tissue for further analyses). The NOMS cohort initially included 25 patients with disease onset between 19 and 40 years of age. This cohort was also expanded by 7 more patients for detailed analysis of remyelination (total *n* = 32, from two patients from the initial cohort we did not have material for further analyses). Inclusion criteria were: (1) histopathological diagnosis of inflammatory demyelinating lesions consistent with MS, (2) disease manifestation at the age ≥ 50 years for the LOMS cohort or 18–40 years for the NOMS cohort, (3) no other confounding brain pathology. Patients with histopathological or serological evidence of neuromyelitis optica spectrum disorders (NMOSD) or acute disseminated encephalomyelitis (ADEM) according to published criteria were excluded from the study [5, 50].

### Neuropathological assessment

Paraffin blocks of brain biopsies were sent to us from other neuropathological centers for a second opinion. The standard procedure for processing human biopsies involved short-term fixation in formalin (24–72 h) before paraffin embedding. Slides were stained with haematoxylin and eosin, Luxol fast blue/periodic acid-Schiff and Bielschowsky’s silver impregnation. Immunhistochemical staining was performed with an avidin–biotin-based method. After tissue preparation, paraffin sections were deparaffinised and rehydrated. Antigen retrieval was performed using either Tris–EDTA (pH 9.0) or citrate buffer (pH 6.0) to unmask epitopes, depending on the antibody. Endogenous peroxidase was blocked with 3% hydrogen peroxide, and non-specific binding was minimised using either fetal bovine serum or normal goat serum. Primary antibodies were applied overnight, followed by biotinylated secondary antibodies and an avidin–biotin-enzyme complex. Finally, a chromogenic substrate (DAB) was used to visualise an antigenic site.

**Fig. 1:**
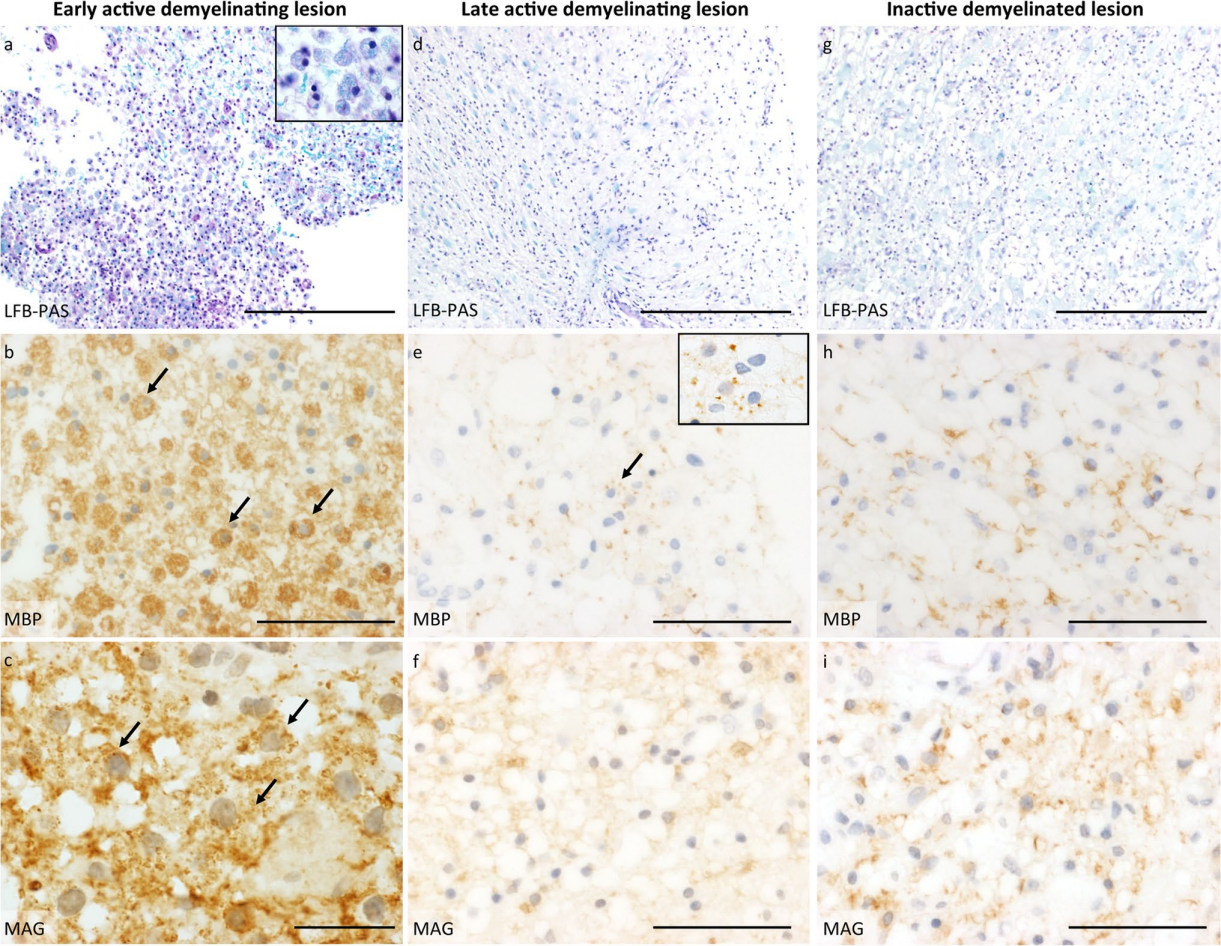
Histopathology of different demyelinating lesion activities. Panels a-c illustrate an **early active demyelinating lesion**, representing the initial stage of demyelination. At this stage, degradation products from both major (e.g., MBP) and minor (e.g., MAG) myelin proteins are present within phagocytes. **a** LFB/PAS staining reveals a demyelinated lesion containing multiple foamy macrophages. The inset shows an enlarged microscopic image of foamy macrophages with LFB-positive myelin debris; **b** Anti-myelin basic protein (MBP) staining of the early active lesion reflects myelin destruction and highlights numerous macrophages filled with myelin degradation products; **c** Anti-myelin-associated glycoprotein (MAG) staining also shows multiple macrophages containing MAG-positive degradation products, a minor myelin protein. Panels **d**-**f** depict a **late active demyelinating lesion**, representing the subsequent stage of demyelination, where only degradation products from major myelin proteins (e.g., MBP) can be identified within phagocytes. **d** LFB/PAS staining shows a completely demyelinated lesion with reactive astrogliosis and foamy macrophages; **e** Anti-MBP staining reflects an advanced stage of demyelination, with only a few macrophages containing MBP-positive degradation products (see inset for MBP-positive macrophages); **f** In contrast, no degradation products from minor myelin proteins, such as MAG, are detected within macrophages, as demonstrated by anti-MAG staining. Panels **g**–**i** present an inactive demyelinated lesion, where no active demyelination processes are observed. **g** LFB/PAS staining reveals a demyelinated lesion accompanied by reactive astrogliosis and foamy macrophages; **h** Anti-MBP staining shows newly formed, thin myelinated fibers, indicating early remyelination. However, no myelin degradation products are observed within macrophages; **i** Anti-MAG staining also identifies early remyelination. *LFB/PAS* Luxol fast blue/periodic acid-Schiff, *MBP* myelin basic protein, *MAG* myelin-associated glycoprotein, Scale bars: **a**, **d** and **g** 200 µm; **b**, **f** and **i** 50 µm; **e** and **h** 100 µm; **c** 20 µm

Two board-certified neuropathologists (I.M. and W.B.) determined the demyelinating activity of the lesions according to published criteria [6]. Early active demyelinating lesions were defined by myelin-laden macrophages immunoreactive for both minor (CNP: 2′3’-cyclic nucleotide 3’phosphodiesterase; MOG: myelin oligodendrocyte glycoprotein; MAG: myelin-associated glycoprotein) and major (MBP: myelin basic protein; PLP: proteolipid protein) myelin proteins (Fig. 1a-c); late active demyelinating lesions are immunoreactive for major myelin proteins, but not for minor myelin proteins (Fig. 1d-f). Inactive demyelinated lesions show no further immunoreactivity within myelin-laden macrophages, neither for major nor for minor myelin proteins (Fig. 1g-i) [6, 19]. These lesion stages indicate the age of demyelinating lesions, with early active demyelinating lesions representing the earliest stages of lesion formation. Non-demyelinated white matter, defined as normal-appearing white matter and periplaque white matter, was also included in the assessment. We defined periplaque white matter as two visual fields (× 40) of non-demyelinated white matter immediately adjacent to the lesion edge. Normal-appearing white matter was defined as non-demyelinated white matter located at least two visual fields from the lesion edge outside the lesion. More than one lesion area (demyelinating activity) and normal-appearing white matter may be present in a single biopsy.

Ninety-six white matter MS lesion areas were analysed. Only biopsies were included, as their lesions typically represent early lesion and also early disease stages. In LOMS patients, 27 lesions were classified as early active demyelinating, 16 as late active demyelinating and 9 as inactive demyelinated. Sixteen areas of normal-appearing white matter were also analysed. Twenty NOMS lesion areas were classified as early active demyelinating, 15 as late active demyelinating, and 9 as inactive demyelinated. In addition, 12 areas of normal-appearing white matter were analysed.

Quantitative histological assessments were then performed. Inflammatory infiltration of the lesions was assessed by immunohistochemical staining for anti-CD3 (all T cells), anti-CD8 (cytotoxic T cells), anti-CD20 (B cells), anti-CD138 (plasma cells), anti-MRP14 (early activated macrophages), and KiM1P (macrophages/microglia). Acute axonal damage was determined by staining with anti-APP (amyloid precursor protein) antibody and the axonal reduction by the Bielschowsky silver staining. Oligodendrocytes were assessed by immunohistochemical staining with anti-Nogo-A, anti-Olig-2 and anti-BCAS1 (breast carcinoma amplified sequence 1) (see Supplementary Table 2 for detailed antibody list). Cell counts were assessed using an ocular morphometric grid. At least five standardized microscopic fields of 62 500µm^2^ each were analysed. Only cells with a nucleus were quantified. The number of APP-positive spheroids was counted in at least three standardized microscopic fields of 10,000 μm^2^ each. All data are expressed as cells or positive axons per mm^2^. Relative axonal density (hereafter referred to as axonal density for simplicity) was determined by point sampling using a 25-point Zeiss eyepiece. Axonal density was expressed as the number of axons crossing the stereological grid points out of a total number of 25 grid points. Measurements were performed using a field size of 95,000 μm^2^ and again, at least three visual fields were evaluated. The median value of cells/APP-positive spheroids and the median axonal density in each patient were used for further statistical analyses. All quantifications were performed blinded to patient age and cross-checked by at least two investigators (LS and SS).

BCAS1 is a marker of early myelinating oligodendrocytes. BCAS1-positive cells can be found in an either quiescent, resting state or an activated, ramified state, indicating active myelination [2]. All lesions were classified into one of the following categories according to the morphological appearance of BCAS1-positive cells: (1) mostly activated: most of the BCAS1-positive cells in lesions show ramified processes as well as a strong positivity of cell bodies; (2) mostly quiescent: lesions with BCAS1-positive cells with expression predominantly on the membrane and within the cytoplasm, but no cell processes can be identified; (3) activated and quiescent: lesions show a combination of activated and quiescent BCAS1-positive cells; (4) and lesions where no BCAS1-positive cells were found (see Fig. 4).

We also analysed twenty-four age-matched autopsy controls who did not have MS. In each case, brain autopsy showed normal findings according to age. Twelve of the autopsy controls were aged between 20 and 40 years at the time of death (defined as younger individuals), while the remaining 12 were over 60 years old (defined as older individuals). Patient demographics as well as associated diseases are listed in Supplementary Table 3. All autopsies were conducted at our institute in accordance with a standard fixation protocol. The fixation duration ranged from two to four weeks.

### Clinical assessment

Clinical information was obtained by review of medical records, as well as through a personal interview and examination. The following clinical data were collected: age at disease onset, age at biopsy, sex, disease course at disease onset and at last follow-up, number of relapses and MS therapies that patients received. Deaths and the cause of the death were also recorded. The Expanded Disability Status Scale (EDSS) score at the time of biopsy and at the last follow-up was noted. The clinical course was defined according to established criteria, namely monophasic, relapsing–remitting, progressive-relapsing, and primary or secondary progressive [40]. The time intervals between symptom onset and biopsy and between biopsy and last follow-up examination were documented.

### Statistical analysis

The data were statistically analysed using GraphPad PRISM® version 6. Descriptive statistics are given for patients in each cohort. A Fisher's exact test was used to compare the global group differences, while a Mann–Whitney U test was applied to assess the histological parameters comparing LOMS and NOMS. A comparison was made between lesions exhibiting the same demyelinating lesion activities. Spearman's correlation and simple linear regression analysis were used to examine the relationship between two parameters. The chi-square test was applied to make a comparison between the morphological subtypes of the lesion with BCAS1-cells. A p-value of 0.05 or less was considered statistically significant. Graphs illustrate the median values with interquartile ranges.

## Results

### Demographic and clinical characteristics of MS cohorts reveal a severe clinical disease course in LOMS

Histopathological analysis was performed on biopsy material from 28 patients with LOMS and 25 patients with NOMS. The demographic and clinical characteristics of the cohorts are summarised in Table 1. No difference in gender distribution was found when comparing the two groups. As we focused on early disease stages with a median disease duration of less than 1 year, most patients in both groups had a monophasic disease course at biopsy. However, the median time interval from biopsy to last follow-up was shorter in LOMS patients. A possible reason for this could be the more severe disease course in LOMS: Eight patients in the LOMS cohort died during the follow-up period, 5 of them from MS. No deaths were observed in the NOMS group. The EDSS at the last follow-up was significantly higher in the LOMS cohort, even after excluding patients who died (p = 0.03). Most patients were not receiving any specific MS treatment at the time of biopsy or were only receiving corticosteroids.

**Table 1:** Clinical and demographic characteristics of LOMS and NOMS patients

	LOMS	NOMS	*p*-value	LOMS extended cohort	NOMS extended cohort	*p*-value extended cohort
Number of patients, n	28	25		31	30	
Females/males, *n* (%)	19/9 (68/32%)	15/10 (60/40%)	0.75	19/12 (61/39%)	18/12 (60/40%)	0.75
Age at time of biopsy, years, median (minimum and maximum)	69 (61–85)	29 (20–35)	** < 0.0001**	68 (51–85)	29 (19–39)	** < 0.0001**
Age at disease onset, years, median (minimum and maximum)	68 (56–85)	28 (17–38)	** < 0.0001**	67 (50–85)	28 (17–39)	** < 0.0001**
Time from disease manifestation to biopsy, days, median (minimum and maximum)	30.5 (6–150)	19 (3–419)	0.71	26 (0–706)	2.4 (0.1–204)	**0.03**
Disease course at biopsy	Monophasic: 16 Relapsing–remitting: 9 Primary progressive: 3	Monophasic: 16 Relapsing–remitting: 8 Primary progressive: 0 Unknown: 1		Monophasic: 18 Relapsing–remitting: 10 Primary-progressive: 3 Unknown: 0	Monophasic: 16 Relapsing–remitting: 11 Primary-progressive: 2 Unknown: 1	
Disease course at last follow-up, n	Monophasic: 16 Relapsing–remitting: 9 Primary progressive: 3	Monophasic: 13 Relapsing–remitting: 10 Primary progressive: 0 Secondary progressive: 1 Unknown: 1		Monophasic: 14 Relapsing–remitting: 11 Primary-progressive: 2 Secondary-progressive:1 Unknown: 3	Monophasic: 10 Relapsing–remitting: 13 Primary-progressive: 1 Secondary-progressive:4 Unknown: 2	
Time from index attack to last follow-up, days, median (minimum and maximum)	441.5 (18–2584)	801 (84–1952)	**0.04**	620.5 (36.5–2701)	1095 (73–5292.5)	**0.04**
Disease duration at last follow-up, days, median (minimum and maximum)	810.3 (41–2773)	1003.75 (84–2132)	0.32	949 (36.5–5730.5)	1606 (73–6935)	0.2
Death; n	8	0	**0.004**	9	0	**0.001**
Causes of death	**MS *n* = 5** Cancer n = 1 Unknown n = 2			MS *n* = 5 Cancer *n* = 2 Unknown *n* = 2		
Number of relapses at last follow-up, median (minimum and maximum)	1 (1–8)	1 (1–10)	0.37	1 (1–8)	2 (1–30)	
EDSS at biopsy, median (minimum and maximum)	3.5 (0–8.5)	3.0 (1,5–9.5)	0.09	3.5 (3.0–8.5)	3.5 (1.0- 9.5)	0.3
EDSS at last follow-up, median (minimum and maximum)	3.5 (0–10)	2.0 (0–9.5)	**0.003**	3.5 (2.5–10.0)	3.0 (1.0–9.5)	**0.05**
EDSS at last follow-up after exclusion of patients that died, median (minimum and maximum)	3.25 (0–9.5)	2.0 (0–9.5)	**0.03**	3.25 (0–9.5)	2.0 (0–9.5)	**0.03**
Therapy prior biopsy	Corticosteroids *n* = 6 Azathioprine (discontinued 3 months before biopsy) *n* = 1 No therapy *n* = 18 Unknown *n* = 3	Corticosteroids *n* = 4 Plasmapheresis *n* = 1 Interferon-beta *n* = 1 No therapy *n* = 17 Unknown *n* = 2		Corticosteroids *n* = 7 Azathioprine (discontinued 3 month for biopsy) n = 1 No therapy *n* = 19 Unknown *n* = 1	Corticosteroids *n* = 7 Plasmapheresis *n* = 1 Interferon-beta *n* = 1 No therapy *n* = 21	
Brain biopsy site	Supratentorial: 27 Infratentorial: 0 Brain stem: 1 Spinal: 0	Supratentorial: 24 Infratentorial: 0 Brain stem: 1 Spinal: 0		Supratentorial: 31 Infratentorial: 0 Brain stem: 1 Spinal: 1	Supratentorial: 29 Infratentorial: 0 Brain stem: 1 Spinal: 0	

Bold values indicate statistically significant differences with a p-value of ≤ 0.05

*MS* multiple sclerosis, *LOMS* late-onset multiple sclerosis, *NOMS* normal-onset multiple sclerosis, *n* number, *EDSS* expanded disability status scale

### No major differences in inflammatory infiltration between LOMS and NOMS in active demyelinating lesions, but increased numbers of T cells and macrophages with age in inactive lesions

The primary objective was to determine whether there were quantitative differences in the inflammatory infiltrates in lesions comparing patients with LOMS and NOMS. The numbers of T cells (CD3), cytotoxic T cells (CD8), B cells (CD20), plasma cells (CD138), microglia/macrophages (KiM1P), and early activated macrophages (MRP14) were quantified within MS lesions of different demyelinating stages and the normal-appearing white matter of LOMS and NOMS patients. No major differences in the infiltration of these cell populations were observed between the two cohorts (Supplementary Figs. 1 and 2, Supplementary Table 4). The number of B cells was lower in the LOMS cohort only in early active lesions (*p* = 0.04) (Supplementary Fig. 2c). We also examined all patients, regardless of cohort, for a possible correlation between age and inflammatory infiltrates. Our results showed that in inactive MS lesions, higher numbers of total T cells (CD3-positive), cytotoxic T cells (CD8-positive), and KiM1P-positive macrophages were associated with older age (CD3-positive T cells: r = 0.7, *p* = 0.04; CD8-positive cytotoxic T cells: r = 0.8, *p* = 0.03 and KiM1P-positive macrophages: r = 0.8, *p* = 0.03; Supplementary Table 5). However, it is important to note that the analysis was based on only eight inactive lesions.

### No major differences in the axonal density and acute axonal injury between LOMS and NOMS, but more pronounced axonal damage in active demyelinating lesions associated with younger age

Second, we sought to determine whether there were differences in the axonal density and acute axonal injury between LOMS and NOMS. To this end, we assessed the density of axons in MS lesions and normal-appearing white matter using Bielschowsky silver staining. Acute axonal injury was evaluated by immunohistochemical staining for amyloid precursor protein (APP). The median axonal density showed no major differences between the two groups of patients (Supplementary Fig. 3a and Supplementary Table 5). A lower axonal density was observed in the NOMS cohort in late active demyelinating lesions (*p* = 0.02). However, no association with patient age was found in the correlation analyses (r = 0.7, *p* = 0.13). In addition, no significant differences were found in acute axonal injury at different MS lesion stages or in normal-appearing white matter when comparing LOMS and NOMS patients (Supplementary Fig. 3b, Supplementary Table 5). Next, correlation analyses between patient age and axonal density or acute axonal injury showed that older age at disease onset was associated with a higher axonal density (r = 0.3, *p* = 0.04) in early active demyelinating MS lesions. Conversely, younger age at disease onset was associated with a more pronounced acute axonal injury (r = − 0.3, *p* = 0.02) in late active demyelinating MS lesions (Supplementary Table 5).

### Mature oligodendrocytes are reduced in the normal-appearing white matter of LOMS patients as well as in older healthy individuals

The number of oligodendrocytes and their precursor cells was then determined in LOMS and NOMS patients using anti-Nogo-A and anti-Olig-2 staining. Anti-Nogo-A is a marker for mature oligodendrocytes, whereas anti-Olig-2 staining detects both oligodendrocyte precursor cells and mature oligodendrocytes. However, a strong nuclear Olig-2 staining is indicative of oligodendrocyte precursor cells and only these cells were quantified [20].

**Fig. 2:**
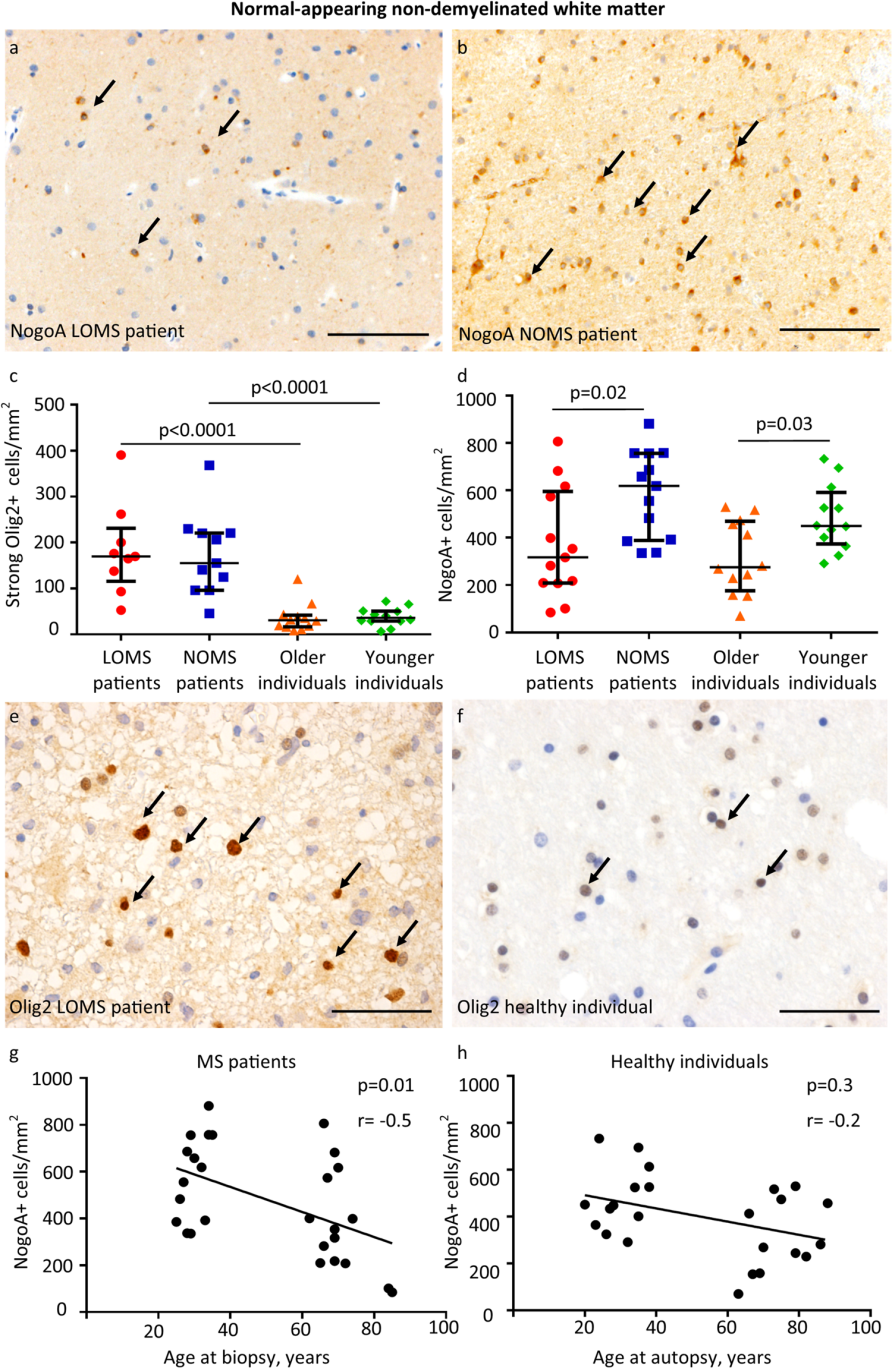
Oligodendrocyte populations in the normal-appearing white matter of MS patients and white matter of healthy individuals **a** A median of 316.8 NogoA-positive mature oligodendrocytes per mm^2^ is present in the normal-appearing, non-demyelinated white matter of LOMS patients (anti-NogoA). **b** In contrast, significantly higher numbers (median of 618.7 NogoA-positive mature oligodendrocytes per mm^2^) are found in the normal-appearing white matter of NOMS patients (anti-NogoA). **c** The number of strongly Olig2-positive oligodendrocyte progenitor cells is increased in the normal-appearing white matter of MS patients compared to healthy individuals (*p* < 0. 0001), with no significant age-related differences. **d** In contrast, a significant reduction in the number of NogoA-positive mature oligodendrocytes is observed in the normal-appearing white matter of LOMS compared to NOMS patients (*p* = 0. 02), as well as in older compared to younger healthy individuals (*p* = 0.03). **e** Anti-Olig2 staining of the non-demyelinated white matter of an LOMS patient: Multiple strongly Olig2-positive nuclei (arrows), corresponding to oligodendrocyte precursors, as well as weakly positive nuclei are seen. **f** Anti-Olig2 staining of the white matter of a healthy older individual: Both strongly (arrows) and weakly Olig2-positive nuclei are identified. **g** A negative correlation between the number of NogoA-positive mature oligodendrocytes and patient age at the time of biopsy is observed (r =− 0.5, *p* = 0.01). **h** No significant correlation is found between the number of NogoA-positive mature oligodendrocytes and the age of healthy individuals (r = − 0.2, *p* = 0.3). **a** and **b** Scale bar = 100µm; **c** and **d** Error bars represent the median with interquartile range; **e** and **f** Scale bar = 50µm; **g** and **h** Non-parametric Spearman correlation index is shown. *LOMS* late-onset multiple sclerosis, *NOMS* normal-onset multiple sclerosis

The number of oligodendrocyte precursor cells did not differ between the two cohorts (Fig. 2c). However, the number of NogoA-positive mature oligodendrocytes was significantly lower in the normal-appearing white matter of LOMS patients (*p* = 0.02, Fig. 2a-b, d). Consistently, correlation analyses showed that lower numbers of mature oligodendrocytes in the normal-appearing white matter were associated with an older patient age (r = − 0.5; *p* = 0.01, Fig. 2g).

To evaluate whether the age-related decline in mature oligodendrocytes in normal-appearing white matter is MS-specific or a general phenomenon associated with ageing, we examined 24 age-matched autopsy controls who did not have MS. The number of oligodendrocyte precursor cells was significantly higher in MS patients compared to age-matched healthy individuals (*p* < 0.0001, Fig. 2c, e–f), indicating proliferation of oligodendrocyte precursor cells during MS. No significant differences in the number of oligodendrocyte precursor were observed between older and younger healthy individuals. Notably, the number of NogoA-positive mature oligodendrocytes did not differ between healthy individuals and MS patients. However, as in MS patients mature oligodendrocytes were significantly lower in older compared to younger healthy individuals (*p* = 0.03, Fig. 2d), but showed no significant correlation with age (r = − 0.2, *p* = 0.3, Fig. 2h).

In summary, our data showed minor differences between LOMS and NOMS patients with respect to inflammatory infiltration, axonal density, and acute axonal injury, which did not show major differences in group comparisons. In contrast, the number of mature oligodendrocytes in the normal-appearing white matter was significantly lower in LOMS patients, with no significant differences found in the number of oligodendrocyte precursor cells. As the same age effect was observed in healthy individuals, we asked whether older patients might have an impaired differentiation of oligodendrocyte precursors into myelinating oligodendrocytes and thus a lower remyelination potential compared to younger individuals.

### Reduced number of early myelinating oligodendrocytes in LOMS

To address this question, we examined the expression of BCAS1. This is a recently well-characterised marker that is transiently expressed in a unique population of early myelinating oligodendrocytes [2, 13]. Previous studies have shown that activated BCAS1-positive oligodendrocytes are found early in lesion formation amidst ongoing inflammation with high numbers of macrophages/microglial cells [2]. To gain insight into the dynamic aspects of remyelination within lesions with different stages of demyelinating activity, we extended our study and included additional cases in each cohort. The demographic and clinical characteristics of the extended cohort are summarised in Table 1. The time interval from the disease manifestation to biopsy was significantly longer in the extended LOMS cohort, whereas the time interval between the attack leading to biopsy and the last follow-up was longer in the extended NOMS cohort.

**Fig. 3:**
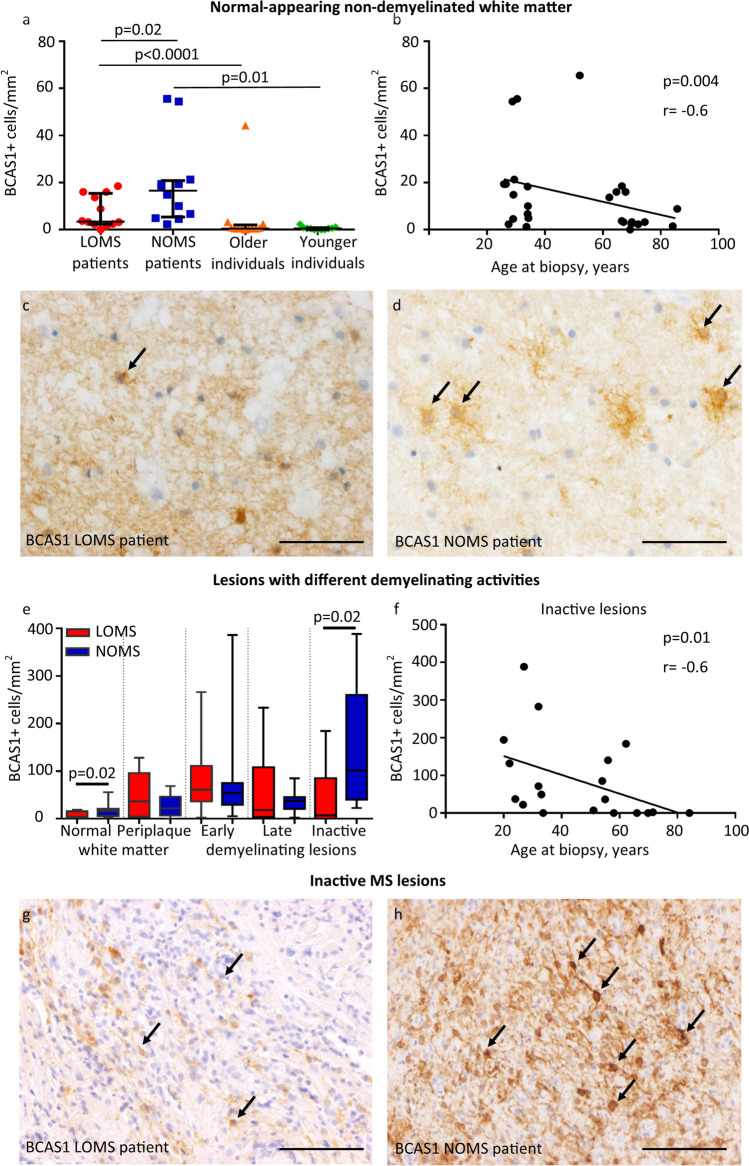
BCAS1-positive early myelinating oligodendrocytes in MS patients and healthy individuals **a** The number of BCAS1-positive early myelinating oligodendrocytes is increased in the normal-appearing, non-demyelinated white matter of MS patients compared to age-matched controls (*p* < 0.0001 for the older cohort and *p* = 0.01 for the younger cohort), and is significantly lower in LOMS compared to NOMS patients (*p* = 0.02). **b** In MS patients, the number of BCAS1-positive early myelinating oligodendrocytes in the normal-appearing white matter shows a negative correlation with patient age at biopsy (r = − 0.6, *p* = 0.004). **c** A median of 3.4 BCAS1-positive cells/mm^2^ is present in the normal-appearing white matter of LOMS patients (arrows). d: In contrast, significantly higher numbers of BCAS1-positive cells (median 16.5 cells/mm^2^) are found in the normal-appearing white matter of NOMS patients (arrows). **e** The number of BCAS1-positive early myelinating oligodendrocytes within lesions with different demyelinating activities, indicating lesion evolution, is shown. BCAS1-positive cells are significantly lower in the normal-appearing white matter and in inactive lesions of LOMS compared to NOMS patients (*p* = 0.02 for both regions). A biphasic increase in BCAS1-positive early myelinating oligodendrocytes is observed in NOMS patients, with an initial rise in active lesions and a second increase in inactive lesions. In contrast, LOMS patients show a further decrease in inactive lesions. **f** A lower number of BCAS1-positive early myelinating oligodendrocytes in inactive MS lesions is associated with an older age of patients (r =− 0.6, *p* = 0.01). **g** Single BCAS1-positive cells are observed in an inactive lesion of a LOMS patient (median: 7.3 cells/mm^2^). **h** Multiple BCAS1-positive cells are present in an inactive lesion of a NOMS patient (median: 101.7 cells/mm^2^). **a** Error bars represent the median with interquartile range; **b** and **f** Non-parametric Spearman correlation index is shown; **c**, **d**, **g** and **h**: Scale bar: 50µm; **e** box plot showing the median number of cells with 10th–90th percentile; whiskers indicate minimum and maximum values. *LOMS* late-onset multiple sclerosis, *NOMS* normal-onset multiple sclerosis

First, the number of all BCAS1-positive oligodendrocytes was examined in healthy controls. Numbers were very low in the white matter (median: 0.5 cells/mm^2^) and no difference was observed between younger and older individuals (*p* = 0.4, Fig. 3a). However, the number of BCAS1-positive oligodendrocytes was significantly higher in the normal-appearing white matter of MS patients in both LOMS and NOMS compared to healthy controls, indicating a general activation of remyelination processes in MS patients (*p* = 0.01 and *p* < 0.0001, respectively, Fig. 3a).

Next, we compared the number of BCAS1-positive oligodendrocytes in the normal-appearing white matter of LOMS and NOMS patients and found significantly lower numbers in the LOMS cohort (*p* = 0.02, Fig. 3a, c-d). Correspondingly, correlation analyses showed that an older age was associated with lower numbers of BCAS1-positive oligodendrocytes in MS patients (r = − 0.6, *p* = 0.004, Fig. 3b).

We then quantified the number of BCAS1-positive oligodendrocytes within the lesions, taking into account the different lesion activities indicating lesion evolution, as well as in the surrounding periplaque white matter (Fig. 3e). In lesions with early active demyelination, representing the earliest lesion stages, the number of BCAS1-positive cells was similar between the LOMS and NOMS cohorts. In contrast, in late active lesions, where demyelination is more advanced, the number of BCAS1-positive oligodendrocytes was lower in the lesions of LOMS compared to NOMS patients, but these differences did not reach statistical significance (median LOMS 21.2 vs NOMS 33.2 cells/mm^2^, *p* = 0.6, Fig. 3e). Finally, the differences in the number of BCAS1-positive oligodendrocytes between LOMS and NOMS peaked in inactive lesions, representing even later lesion stages. The number of BCAS1-positive oligodendrocytes in inactive lesions was significantly lower in LOMS patients (median LOMS 4.8 vs NOMS 71.3 cells/mm^2^, *p* = 0.02, Fig. 3e, g-h). Furthermore, the number of BCAS1-positive oligodendrocytes in inactive lesions showed a negative correlation with patient age (r = − 0.6, *p* = 0.01, Fig. 3f). Interestingly, the dynamics of BCAS1 expression differed between patient cohorts: In NOMS patients we observed a biphasic increase of BCAS1-positive cells in early active and inactive lesions, whereas in LOMS patients, after an initial increase in early active lesions, BCAS1-positive cells continuously decreased with lesion progression (Fig. 3e).

### Morphological evaluation indicates impaired remyelination in LOMS lesions

It has been previously described that the morphological features of oligodendrocytes reflect the function they are currently performing [2, 9, 13, 21, 42]. Here we also observed that BCAS1-positive cells exhibited distinct morphological features, with some showing positive cell bodies and numerous ramified processes, suggesting an active myelinating morphology, termed activated BCAS1-positive oligodendrocytes. In contrast, other cells showed BCAS1 expression on the cell membrane and within the cytoplasm with no prominent processes, suggesting an inactive or progenitor morphology, termed quiescent BCAS1-positive oligodendrocytes.

**Fig. 4:**
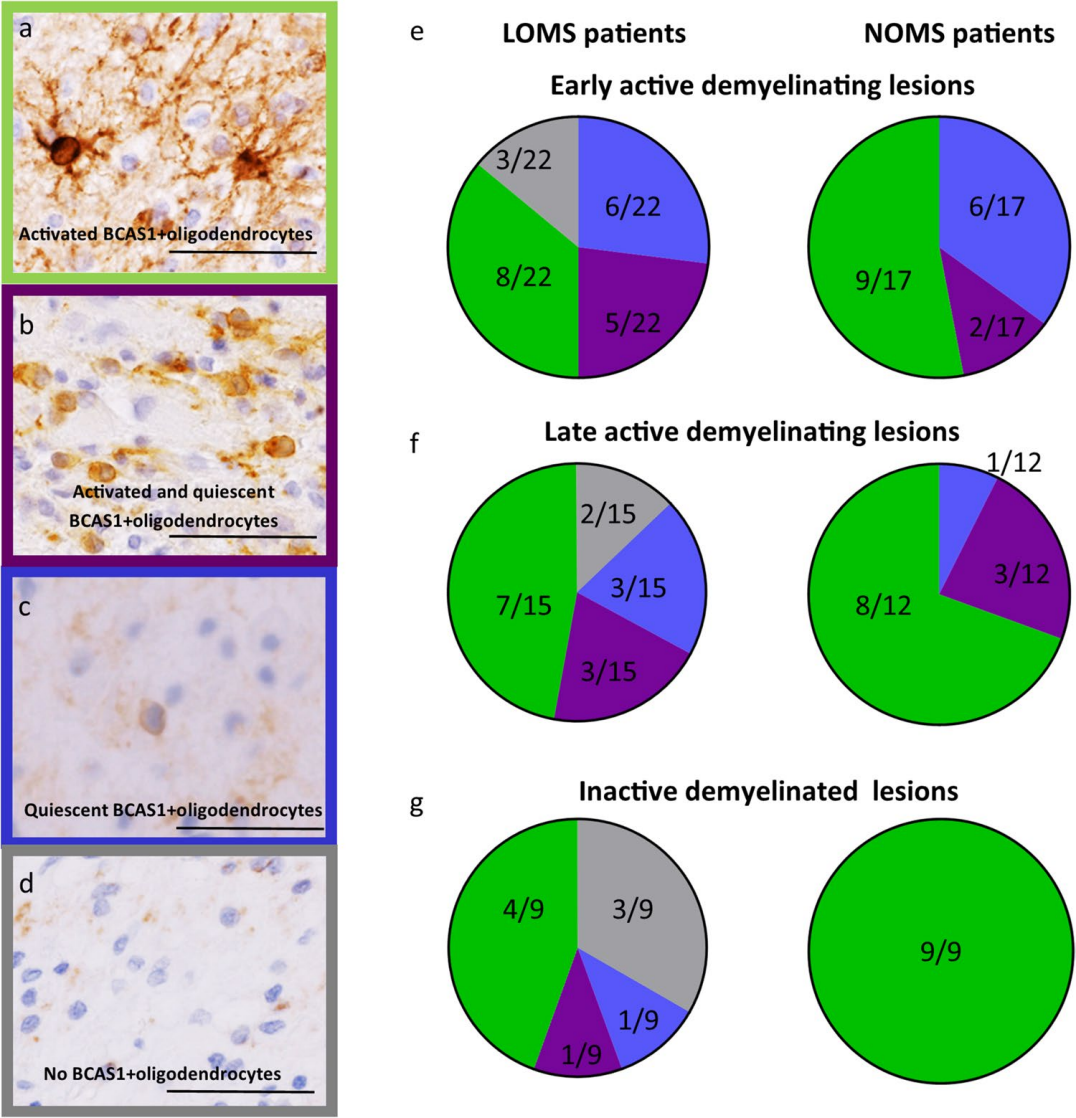
Distribution of BCAS1-positive oligodendrocytes with resting and activated morphologies in MS lesions with different demyelinating activities **a** BCAS1-positive cells with strongly positive cell bodies and ramified processes are shown, referred to as *activated BCAS1-positive oligodendrocytes*. **b** Lesions with BCAS1-positive cells showing strongly positive cell bodies and ramified processes, as well as cells with BCAS1 expression only on the cell membrane and within the cytoplasm, referred to as *activated and quiescent BCAS1-positive oligodendrocytes,* are depicted. **c** BCAS1-positive cells with expression predominantly on the cell membrane and within the cytoplasm, but not showing ramified processes, are found in this case, referred to as *quiescent BCAS1-positive oligodendrocytes*. **d** Finally, some lesions show *no BCAS1-positive* cells. **e** 53% of early active demyelinating lesions in NOMS patients show mostly activated BCAS1-positive oligodendrocytes, whereas the proportion is lower in LOMS (36%). The percentage of lesions with mostly quiescent BCAS1-positive oligodendrocytes and lesions with both activated and quiescent BCAS1 + oligodendrocytes does not differ significantly between groups. However, a small percentage (14%) of lesions without any BCAS1-positive cells is observed only in LOMS. **f** In late active demyelinating lesions, the percentage of lesions with mostly activated BCAS1-positive cells increases in both cohorts, reaching 75% of lesions in NOMS and 47% in LOMS. The presence of lesions with mostly quiescent BCAS1-positive cells decreases in both cohorts, but the reduction is more pronounced in NOMS, from 35 to 8%, and less pronounced in LOMS, from 27 to 20%. Lesions without any BCAS1-positive cells are present only in LOMS (13%). **g** Inactive demyelinated lesions of NOMS patients show only lesions with activated BCAS1-positive cells (100%). In LOMS, the percentage of these lesions does not change compared to late active demyelinating lesions (44%). One in three lesions in LOMS patients have no BCAS-1 cells at all (33%). **a**–**d**: Scale bar = 50µm. *LOMS* late-onset multiple sclerosis, *NOMS* normal-onset multiple sclerosis

We evaluated the morphological characteristics of BCAS1-positive cells in MS lesions by distinguishing four lesions subtypes: lesions in which the majority of BCAS1-positive cells showed an activated morphology (Fig. 4a); lesions with BCAS1-positive cells in which the majority of cells showed a quiescent morphology (Fig. 4c); lesions in which both of the cell morphologies were present (activated and quiescent, Fig. 4b), and lesions in which no BCAS1-positive cells were found (Fig. 4d). We investigated the distribution of these morphological lesion subtypes in lesions with different demyelinating activities and compared the LOMS and NOMS cohorts.

Half of the early active demyelinating lesions in NOMS patients showed predominantly activated BCAS1-positive oligodendrocytes, whereas the proportion of these lesions was lower in LOMS (36%, Fig. 4e, shown in green). The percentage of lesions with both cell morphologies (purple) and lesions with mostly quiescent BCAS1-positive oligodendrocytes (blue) was similar between LOMS and NOMS patients, but a small percentage (14%) of lesions without any BCAS1-positive cells was found only in LOMS (grey, Fig. 4e). Analysis of the next stage of lesion evolution, late active demyelinating lesions, showed that the proportion of lesions with predominantly activated BCAS1-positive cells increased in both cohorts, reaching 75% of lesions in NOMS and 47% in LOMS (Fig. 4f). At the same time, the presence of lesions with mostly quiescent BCAS1-positive cells decreased in both cohorts, but the reduction was significantly higher in the NOMS cohort, from 35 to 8%, whereas in LOMS the reduction was more modest, from 27 to 20%. Again, lesions without any BCAS1-positive cells were present only in LOMS (Fig. 4f). The most dominant differences were observed in inactive demyelinated lesions. Here, all lesions in NOMS patients were characterised by the presence of mostly activated BCAS1-positive cells (Fig. 4g). In LOMS, the percentage of these lesions did not change much compared to late active demyelinating lesions (44%). One of three lesions in LOMS patients had no BCAS1-positive cells at all (33%, Fig. 4g).

The analysis of early and late active demyelinating lesions revealed no significant differences in morphological subtypes between the LOMS and NOMS cohorts (*p* = 0.3 and *p* = 0.4, respectively, chi-squared test). However, in inactive lesions, despite the smaller number of patients, there was a clear tendency toward differences between the two groups. While this analysis did not reach statistical significance (*p* = 0.07), further investigation may provide more conclusive results.

Thus, our study demonstrates that LOMS patients are characterised by a reduced number of mature and early myelinating oligodendrocytes in the normal-appearing white matter and by limited myelinating activity in lesions compared with NOMS patients. Finally, we asked whether these histological changes correlated with patients` clinical disability.

### Higher disability score associated with fewer oligodendrocytes in LOMS

**Fig. 5:**
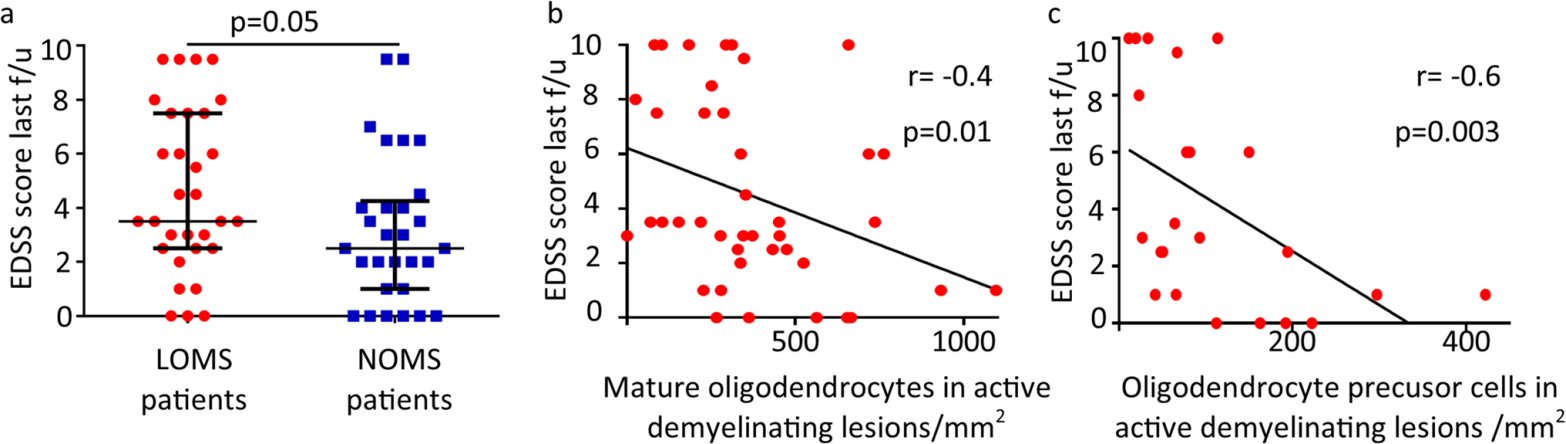
Correlation of mature oligodendrocytes and oligodendrocyte precursor cells with the EDSS score at the last follow-up **a** The EDSS score at the last follow-up was significantly higher in LOMS patients compared to NOMS patients (median: LOMS 3.5 vs. NOMS 2.0, *p* = 0.05). **b** A lower number of NogoA-positive mature oligodendrocytes is associated with a higher disability score at the last follow-up, as measured by the EDSS (r =− 0.4, *p* = 0.01). c: A Lower number of strongly Olig2-positive oligodendrocyte precursor cells is associated with a higher EDSS score at the last follow-up (r = − 0.6, *p* = 0.003). **a** Error bars represent the median with interquartile range; **b** and **c**: Non-parametric Spearman correlation index is shown**.** *LOMS* late-onset multiple sclerosis, *NOMS* normal-onset multiple sclerosis, *EDSS* Expanded disability status scale

It is generally accepted that remyelination protects axons from damage and promotes neurological recovery after relapses. In our study, LOMS patients had a higher disability score measured by EDSS at the last follow-up (median 3.5) compared to NOMS patients (median 2.5, *p* = 0.05, Fig. 5a). We correlated the number of oligodendrocytes of different oligodendrocyte populations with the patients´ disability scores. A negative correlation was found for mature oligodendrocytes (r = − 0.4, *p* = 0.01, Fig. 5b) and oligodendrocyte progenitor cells (r = − 0.6, *p* = 0.003, Fig. 5c) in active demyelinating MS lesions. BCAS1-positive cells showed no association with EDSS at the last follow-up (Supplementary Table 6).

## Discussion

In recent decades, there has been a noticeable increase in the average age of MS disease onset [31], emphasizing the clinical relevance of this group of patients. LOMS patients differ from NOMS patients in their clinical characteristics, with more patients initially presenting with a primary progressive disease course, a lower recovery rate from relapses and a higher level of disability (see also Supplementary Table 1; [10, 25, 32, 33]). Preventing the accumulation of permanent neurological disability is the main goal in the management of MS patients, and slowing down the accumulation of disability with modern therapeutics represents an important milestone in MS therapy. In the present study, we investigated the histopathological features of LOMS patients and compared them with lesions of NOMS patients. Our quantitative analyses demonstrated that the inflammatory infiltration, axonal density, and acute axonal injury did not show major differences from those observed in NOMS patients. However, significant differences were observed when analysing different oligodendrocyte subpopulations: The numbers of mature NogoA-positive oligodendrocytes as well as BCAS1-positive early myelinating oligodendrocytes were reduced in the normal-appearing white matter of LOMS patients, and both cell populations showed a negative correlation between patient age and the number of oligodendrocytes. Furthermore, the number of BCAS1-positive oligodendrocytes was reduced in inactive MS lesions in LOMS, and almost half of the lesions showed morphological changes suggesting impaired remyelination, with one-fifth of patients showing no BCAS1-positive cells at all. In contrast, BCAS1-positive cells with a ramified morphology were observed in all inactive lesions of NOMS patients, suggesting an active remyelination.

Importantly, our study focused on biopsy samples from patients with an early disease stage (median disease duration of 30 days for LOMS and 19 days for NOMS patients). This allowed us to specifically identify age-dependent effects on MS histopathology, as opposed to effects due to a longer disease course. Previous studies of human MS tissue have shown that oligodendrocyte precursor cells and mature oligodendrocytes are not significantly reduced in the early stages of the disease in the general MS population, which is not stratified by age at disease onset [7, 15, 23]. In late stages of the disease, which are often characterized by a progressive disease course, histopathology shows fundamental differences compared with early stages, including reduced oligodendrocyte numbers and less efficient remyelination, highlighting that multiple factors influence remyelination efficiency [7, 20, 45].

Our data suggest, that the most striking histopathological differences between LOMS and NOMS are found in the oligodendrocyte populations and their remyelination efficacy. Oligodendrocytes are the myelin-maintaining cells in the CNS. Both rodent and human in vitro cell culture studies have unraveled the differentiation steps of human oligodendrocyte precursor cells into myelinating oligodendrocytes [1, 14]. During remyelination, oligodendrocyte precursor cells appear to be recruited from the surrounding normal-appearing white matter, as MS lesions are typically characterised by an increased density of oligodendrocyte precursor cells in the periplaque white matter, as was also shown in the present study [20]. The higher number of oligodendrocyte precursor cell in the normal-appearing white matter was independent of the age of the patients. Our study also showed that BCAS1-positive oligodendrocytes, which represent the next stage of differentiation, were increased in the normal-appearing white matter of MS patients. However, the number of these cells was significantly lower in patients with LOMS compared to NOMS, indicating an age-related impairment in the differentiation of oligodendrocyte precursors. This hypothesis was further corroborated by the lower number of mature oligodendrocytes in both LOMS patients and older healthy controls.

However, remyelination can occur not only through the proliferation and differentiation of newly generated oligodendrocytes but also through pre-existing oligodendrocytes [22, 48, 49]. With the reduction of mature oligodendrocytes observed in the elderly population, this remyelination pathway may also play a role in a less effective remyelination in LOMS.

The impact of age on normal myelination and remyelination has been elucidated in animal studies [36, 37]. In remyelination experiments with aged mice, a delay in the recruitment and differentiation of oligodendrocyte precursor cells was observed [35, 37, 46]. However, increasing the number of oligodendrocyte precursor cells had no effect on the remyelination rate, suggesting that insufficient remyelination in aged mice is likely due to defects in oligodendrocyte differentiation [47], which is consistent with our findings. In the present study, we observed that, although oligodendrocyte precursor cell numbers did not differ between LOMS and NOMS patients, the proportion of lesions with ramified, active myelinating BCAS1-positive oligodendrocytes was significantly reduced in LOMS patients. This may be interpreted as further evidence of an aged-related block in oligodendrocyte differentiation. Moreover, lesions completely devoid of BCAS1-positive oligodendrocytes were observed only in LOMS patients, indicating that these lesions do not harbor remyelination potential from differentiating oligodendrocytes. Therefore, our results suggest that agents with the potential to stimulate oligodendrocyte differentiation should be added to the therapy in LOMS from the onset of the disease to promote remyelination potential. However, these therapeutic approaches must take into account the complex and dynamic tissue environment that influences remyelination.

Both physiological ageing and inflammation-induced accelerated cellular senescence contribute to oligodendroglial differentiation blockade and remyelination failure in MS [34, 35, 38, 44]. Age-related epigenetic changes in histone deacetylation and methylation contribute to the expression of remyelination inhibitory factors [35]. Furthermore, oligodendrocyte progenitor cells isolated from old animals show reduced differentiation in cell culture and do not respond to factors that normally force differentiation of cells isolated from young animals [28, 34]. Further studies showed that in particular the terminal differentiation of rodent oligodendrocytes into mature myelinating oligodendrocytes is affected by age. However, after transplantation of oligodendrocyte precursor cells from old mice into the brains of newborn rats, they were able to proliferate and migrate at a rate comparable to that of transplanted neonatal control cells [34, 44], emphasizing the role of the microenvironment for oligodendrocyte precursor cell differentiation. Thus, a senescent immune environment may negatively influence remyelination in patients with LOMS.

To our knowledge, this is the first comprehensive histopathological study of LOMS patients, focusing on early disease stages. This unique cohort allows us to assess age-related effects while excluding the influence of disease duration. Although we cannot entirely exclude prior silent MS lesions, we mitigated this risk through a thorough chart review. Regional differences in remyelination potential were considered, but there was no clear difference in brain regions biopsied between LOMS and NOMS patients (Table 1). Further limitations include potential fixation artifacts, observer variability (addressed through independent verification), sampling bias in small biopsies, and the small sample size of certain lesion subgroups, which may affect statistical analyses.

In conclusion, our study suggests that remyelination is impaired in patients with LOMS compared to patients with NOMS. Mature oligodendrocytes were reduced in normal-appearing white matter, whereas early myelinating oligodendrocytes were reduced in normal-appearing white matter and in inactive MS lesions of LOMS patients. Morphological studies further corroborated an impaired remyelination in lesions from LOMS patients. The reduction in oligodendrocytes was associated with a higher neurological disability in older patients, underlining the potential clinical significance of these findings. We suggest that future therapeutic studies should exploit therapeutics that promote the differentiation of oligodendrocyte precursor cells into mature myelinating oligodendrocytes, which may be particularly promising in patients with LOMS.

## Supplementary Information

Below is the link to the electronic supplementary material.

Supplementary file1 (DOCX 67 KB)

Supplementary file2 (DOCX 17 KB)

Supplementary file3 (DOCX 14 KB)

Supplementary file4 (DOCX 19 KB)

Supplementary file5 (DOCX 17 KB)

Supplementary file6 (DOCX 12 KB)

Supplementary file7 (TIF 4088 KB)

Supplementary file8 (TIF 3252 KB)

Supplementary file9 (TIF 2991 KB)
