# Supplementary material for: Impaired remyelination in late-onset multiple sclerosis
Source: Acta Neuropathol. 2025 Apr 1;149(1):30. doi: 10.1007/s00401-025-02868-5 (PMC11961469; doi:10.1007/s00401-025-02868-5)
Supplement: Supplementary file 1 — Supplementary file1 (DOCX 67 KB) [file 401_2025_2868_MOESM1_ESM.docx]

Supplementary Table 1: Comparison of clinical, CSF and MRI characteristics of LOMS versus NOMS patients

|  | NOMS | LOMS |
| --- | --- | --- |
| Sex ratio (F:M) | 2:1 to 3:1 [14] | 1.4:1 to 1.7:1[5, 11] |
| Disease course at disease onset | 85% RRMS, 5% PPMS, later conversion to SPMS [5] | 50% RRMS [5], 25-50% PPMS, faster conversion to SPMS [9, 11, 13] |
| Symptoms at disease onset: | | |
| Visual disturbances | 37% opticus neuritis [1, 5] | 5% opticus neuritis [5, 11] |
| Motor dysfunction | 29% hemi/paraparesis [3, 5, 6] | 63-100% paraparesis of the lower extremities [5, 6, 10, 11, 13] |
| Cerebellar symptoms | 12% ataxia [5] | 25% ataxia [5, 13] |
| Sensory dysfunction | 71% paresthesia [4, 5] | 30-94% [5, 10, 11] |
| Bowel and bladder dysfunction | 20% bladder disorders  4.8% bowel disorders [4] | “More frequently as compared to NOMS” [8, 12] |
| Disease progression | Slower progression compared to LOMS [5, 11, 13] | Faster disease progression compared to NOMS [5, 6, 9, 11] |
| Recovery from relapses | 87% significant recovery from relapses [2] | 68% significant recovery from relapses, negative association with age [2] |
| Response to HDCS | 93% response rate [5] | 73% response rate [5] |
| CSF findings | 98% OCB +  67% pleocytosis [5, 7, 10] | 92% OCB +  34% pleocytosis [5] |
| MRI findings | 63% Gd+ lesions  98% supratentorial lesions  62% infratentorial lesions  48% spinal lesions [5] | 15% Gd+ lesions  96% supratentorial lesions  44% infratentorial lesions  60-81% spinal lesions [5, 10] |

F: female; M: male; NOMS: normal-onset multiple sclerosis; LOMS: late-onset multiple sclerosis; RRMS: relapsing-remitting multiple sclerosis; PPMS: primary progressive multiple sclerosis; SPMS: secondary progressive multiple sclerosis; OCB: oligoclonal bands; Gd+: gadolinium enhancement; HDCS: high-dose corticosteroids.

References

1 Amador-Patarroyo MJ, Rodriguez-Rodriguez A, Montoya-Ortiz G (2012) How does age at onset influence the outcome of autoimmune diseases? Autoimmune diseases 2012: 251730 Doi 10.1155/2012/251730

2 Cossburn M, Ingram G, Hirst C, Ben-Shlomo Y, Pickersgill TP, Robertson NP (2012) Age at onset as a determinant of presenting phenotype and initial relapse recovery in multiple sclerosis. Mult Scler 18: 45-54 Doi 10.1177/1352458511417479

3 Garg N, Smith TW (2015) An update on immunopathogenesis, diagnosis, and treatment of multiple sclerosis. Brain and behavior 5: e00362 Doi 10.1002/brb3.362

4 Kip M. ST, Bleß H.-H (2016) Weßbuch Multiple Sklerose. Springer Berlin Heidelberg, City

5 Kis B, Rumberg B, Berlit P (2008) Clinical characteristics of patients with late-onset multiple sclerosis. J Neurol 255: 697-702 Doi 10.1007/s00415-008-0778-x

6 Knowles S, Middleton R, Cooze B, Farkas I, Leung YY, Allen K, Winslade M, Owen DRJ, Magliozzi R, Reynolds Ret al (2024) Comparing the Pathology, Clinical, and Demographic Characteristics of Younger and Older-Onset Multiple Sclerosis. Ann Neurol 95: 471-486 Doi 10.1002/ana.26843

7 Konen FF, Hannich MJ, Schwenkenbecher P, Grothe M, Gag K, Jendretzky KF, Gingele S, Sühs KW, Witte T, Skripuletz Tet al (2022) Diagnostic Cerebrospinal Fluid Biomarker in Early and Late Onset Multiple Sclerosis. Biomedicines 10: Doi 10.3390/biomedicines10071629

8 Martinelli V, Rodegher M, Moiola L, Comi G (2004) Late onset multiple sclerosis: clinical characteristics, prognostic factors and differential diagnosis. Neurological sciences : official journal of the Italian Neurological Society and of the Italian Society of Clinical Neurophysiology 25 Suppl 4: S350-355 Doi 10.1007/s10072-004-0339-8

9 Mouresan EF, Mentesidou E, Berglund A, McKay KA, Hillert J, Iacobaeus E (2024) Clinical Characteristics and Long-Term Outcomes of Late-Onset Multiple Sclerosis: A Swedish Nationwide Study. Neurology 102: e208051 Doi 10.1212/wnl.0000000000208051

10 Naseri A, Nasiri E, Sahraian MA, Daneshvar S, Talebi M (2021) Clinical Features of Late-Onset Multiple Sclerosis: a Systematic Review and Meta-analysis. Mult Scler Relat Disord 50: 102816 Doi 10.1016/j.msard.2021.102816

11 Polliack ML, Barak Y, Achiron A (2001) Late-onset multiple sclerosis. Journal of the American Geriatrics Society 49: 168-171 Doi 10.1046/j.1532-5415.2001.49038.x

12 Tobin WO, Costanzi C, Guo Y, Parisi JE, Weigand SD, Lucchinetti CF (2017) Clinical-radiological-pathological spectrum of central nervous system-idiopathic inflammatory demyelinating disease in the elderly. Mult Scler 23: 1204-1213 Doi 10.1177/1352458516675748

13 Tremlett H, Devonshire V (2006) Is late-onset multiple sclerosis associated with a worse outcome? Neurology 67: 954-959 Doi 10.1212/01.wnl.0000237475.01655.9d

14 Voskuhl RR, Gold SM (2012) Sex-related factors in multiple sclerosis susceptibility and progression. Nat Rev Neurol 8: 255-263 Doi 10.1038/nrneurol.2012.43
