# Supplementary material for: Impaired remyelination in late-onset multiple sclerosis
Source: Acta Neuropathol. 2025 Apr 1;149(1):30. doi: 10.1007/s00401-025-02868-5 (PMC11961469; doi:10.1007/s00401-025-02868-5)
Supplement: Supplementary file 2 — Supplementary file2 (DOCX 17 KB) [file 401_2025_2868_MOESM2_ESM.docx]

Supplementary table 2. Antibody list

| **Antibody** | **Clone (number, company)** | **Origin** | **Antigen retrieval** | **Dilution** | **RRDI** | **Target** |
| --- | --- | --- | --- | --- | --- | --- |
| **CD3** | Monoclonal  (SP7, DCS) | Rabbit | Citrate buffer | 1:25 | n.a. | T cells |
| **CD8** | Monoclonal  (C8/144B, Dako) | Mouse | Citrate buffer | 1:50 | AB_2075537 | Cytotoxic T cells |
| **CD20** | Monoclonal  (L26, Dako) | Mouse | none | 1:100 | AB_2282030 | B cells |
| **CD138** | Monoclonal  (MI15, Dako) | Mouse | EDTA buffer | 1:50 | AB_2254116 | Plasma cells |
| **KiM1P** | Monoclonal  (Kiel) | Mouse | Citrate buffer | 1:5000 | n.a. | Macrophages, microglia |
| **MRP14** | Monoclonal  (Acris) | Mouse | Protease | 1:500 | AB_350011 | Early activated macrophages |
| **APP** | Monoclonal  (22C11, Chemicon) | Mouse | Citrate buffer | 1:2000 | AB_94882 | Amyloid precursor protein (acute axonal dammage) |
| **NOGO-A (H-300)** | Polyclonal  (Santa Cruz) | Rabbit | Citrate buffer | 1:500 | AB_2285559 | Mature oligodendrocytes |
| **Olig-2** | Polyclonal  (IBM) | Rabbit | EDTA buffer | 1:150 | AB_1630817 | Oligodendrocytes /oligodendrocyte precusor cells |
| **NaBC1/**  **BCAS1** | Monoclonal  (sc-136342,  Santa Cruz) | Mouse | Citrate buffer | 1:500 | AB_10839529 | Active myelinating oligodendrocytes |
| **MBP** | Polyclonal  (Dako) | Rabbit | None | 1:2000 | AB_2650566 | Myelin basic protein |
| **PLP** | Monoclonal  (pipC1 AbD, Serotec) | Mouse | Citrate buffer | 1:500 | AB_2237198 | Proteolipid protein |
| **MOG** | Monoclonal | Rate | Citrate buffer | 1:1000 | n.a. | Myelin oligodendrocyte glycoprotein |
| **MAG** | Monoclonal  (Abcam) | Mouse | Citrate buffer | 1:1000 | AB_2042411 | Myelin-associated glycoprotein |
| **CNP** | Monoclonal (SMI 91, Covance) | Mouse | Citrate buffer | 1:200 | AB_510038 | Cyclic nucleotide phosphodiesterase |
