# Supplementary material for: Impaired remyelination in late-onset multiple sclerosis
Source: Acta Neuropathol. 2025 Apr 1;149(1):30. doi: 10.1007/s00401-025-02868-5 (PMC11961469; doi:10.1007/s00401-025-02868-5)
Supplement: Supplementary file 3 — Supplementary file3 (DOCX 14 KB) [file 401_2025_2868_MOESM3_ESM.docx]

Supplementary Table 3: Clinical and demographic data of the non-MS healthy control autopsy cases

|  | Older individuals | Younger individuals | p-value |
| --- | --- | --- | --- |
| Number of patients, n | 12 | 12 |  |
| Females/males, n (%) | 3/9 (25/75 %) | 3/9 (25/75 %) | 1.0 |
| Age at time of autopsy, years, median (min- max) | 74 (63-88) | 30 (20-38) | **<0.0001** |
| Cause of death | Heart attack n=5  Hemorrhagic shock after traumatic injury n=1  Unclear n=2  Acute kidney insufficiency n=2  Carbon monoxide poisoning n=1  Asphyxia n=1 | Aneurysmal rupture n=1  Cerebellar bleeding n=1  Unclear n=6  Suicide n=1  Septic shock n=2  Acute liver insufficiency n=1 |  |
| Other comorbidities | Chronic heart failure: n=4  Coronary heart disease n=5  Arterial hypertension n=4  Liver cirrhosis n=3  Alcoholism n=1  COPD n=3  Diabetes mellitus type I n=1  Diabetes mellitus type II n=2  Non-CNS tumors (kidney, lung, intestine) n=2  Chronic kidney insufficiency n=1 | Endocarditis n=2  Epilepsy n=1  Psychiatric disorders n=1  Acute kidney insufficiency n=1  HIV n=1  Liver cirrhosis n=1  Hepatitis C n=1  Hepatitis B n=1  Drug abuse n=2  Polytoxomania n=1  Arterial hypertension n=1  Anorexia n=1  Sepsis n=1  ECMO n=1  Heart insufficiency n=1  Adenocarcinoma n=1  None n=3 |  |

COPD: chronic obstructive pulmonary disease; ECMO: extracorporeal membrane oxygenation
