# Supplementary material for: Impaired remyelination in late-onset multiple sclerosis
Source: Acta Neuropathol. 2025 Apr 1;149(1):30. doi: 10.1007/s00401-025-02868-5 (PMC11961469; doi:10.1007/s00401-025-02868-5)
Supplement: Supplementary file 4 — Supplementary file4 (DOCX 19 KB) [file 401_2025_2868_MOESM4_ESM.docx]

Supplementary Table 4: Quantification of inflammatory cells, axonal density and acute axonal damage in non-demyelinated white matter and lesions with different demyelinating activities in LOMS and NOMS patients

| Cell type (Marker) | Non-demyelinated white matter, median/mm^2^ (min-max) | p-value  (Mann-Whitney test) | Early active demyelinating lesions, median/mm^2^ (min-max) | p-value  (Mann-Whitney test) | Late active demyelinating lesions,  median/mm^2^ (min-max) | p-value  (Mann-Whitney test) | Inactive lesions,  median/mm^2^ (min-max) | p-value  (Mann-Whitney test) |
| --- | --- | --- | --- | --- | --- | --- | --- | --- |
| T cells/mm^2^ (CD3) LOMS | 10.8 (2.4 - 76.8) | 0.06 | 69.3 (0 - 569.6) | 0.53 | 120.1 (22.4 - 454.0) | 0.78 | 238.9 (3.2 - 689.6) | 0.33 |
| T cells/mm^2^ (CD3) NOMS | 4.8 (0.9 - 17.4) |  | 89.2 (21.81 - 370.1) |  | 141.6 (21.6 - 1076) |  | 54.4 (39.2 - 96) |  |
| Cytotoxic T cells/mm^2^ (CD8) LOMS | 9.3 (2.4 - 63.2) | 0.09 | 24.0 (0 - 499.2) | 0.32 | 62.0 (6.6 - 302.5) | 0.8 | 185.4 (28.8 - 248.8) | 0.09 |
| Cytotoxic T cells/mm^2^ (CD8) NOMS | 5.2 (1.6 - 17.6) |  | 36.8 (12.8 - 168.9) |  | 63.2 (10.4 - 433.6) |  | 28.8 (11.2 - 30.4) |  |
| B cells/mm^2^ (CD20) LOMS | 0 (0 - 48.8) | 0.62 | 0 (0 - 59.7) | 0.04 | 0 (0 - 308.8) | 0.56 | 1.5 (0 - 283.2) | 0.39 |
| B cells/mm^2^ (CD20) NOMS | 0 (0 - 3.2) |  | 0.8 (0 - 110.4) |  | 0.4 (0 - 11.4) |  | 0 (0 - 0) |  |
| Plasma cells/mm^2^ (CD138) LOMS | 0 (0 - 2.4) | 0.3 | 1.4 (0 - 157.1) | 0.25 | 3.2 (0 - 282.4) | 0.95 | 8.0 (0 - 268.8) | 0.38 |
| Plasma cells/mm^2^ (CD138) NOMS | 0 (0 - 3.2) |  | 3.2 (0 - 99.2) |  | 3.8 (0 - 58.4) |  | 0 (0 - 8.8) |  |
| Microglia/mm^2^ (KiM1P) LOMS | 194.4 (30.7 - 977.9) | 0.67 | 0 (0 - 80.0) | >0.99 | 0 (0 - 760.8) | 0.59 | 153.6 (0 - 409.6) | 0.32 |
| Microglia/mm^2^ (KiM1P) NOMS | 204.8 (105.6 - 399.2) |  | 0 (0 - 60.8) |  | 0 (0 - 96) |  | 0 (0 - 36.8) |  |
| Macrophages/mm^2^ (KiM1P) LOMS | 0 (0 - 136) | 0.44 | 1379 (859.2 - 2053) | 0.43 | 1208 (652.8 - 2885) | 0.95 | 1026 (800 - 1904) | 0.25 |
| Macrophages/mm^2^ (KiM1P) NOMS | 0 (0 - 37.3) |  | 1603 (644.8 - 2497) |  | 1129 (694.4 - 2325) |  | 825.6 (786.4 - 1006) |  |
| Early activated macrophages/mm^2^ (MRP14) LOMS | 0.8 (0 - 8.8) | 0.14 | 331 (0 - 1415) | 0.39 | 29.1 (0 - 160.8) | 0.37 | 19 (0 - 62.4) | 0.28 |
| Early activated macrophages/mm^2^ (MRP14) NOMS | 1.6 (0 - 17.6) |  | 508.6 (0 - 1510) |  | 52 (5.3 - 396.8) |  | 2 (0 - 8.0) |  |
| Axonal spheroids/ acute axonal damage/mm^2^ (APP) LOMS | 10 (0 - 220) | 0.5 | 470 (25 - 1940) | 0.68 | 190 (0 - 909) | 0.55 | 180 (30 - 245) | 0.78 |
| Axonal spheroids/acute axonal damage/mm^2^ (APP) NOMS | 13.3 (0 - 120) |  | 830 (27 - 2365) |  | 342 (40 - 1345) |  | 210 (5 - 506.2) |  |
| Axonal density (Bielschowsky silver staining), % of axons relative to non-demyelinated white matter LOMS | |  | 55.4% (28.7 - 80.6%) | 0.74 | 66.2% (34.3 - 80%) | 0.02 | 57.2% (46.5 - 77%) | 0.13 |
| Axonal density (Bielschowsky silver staining), % of axons relative to non-demyelinated white matter NOMS | |  | 52.6% (27.4 - 83.8%) |  | 52.6% (26.8 - 69.2%) |  | 31.3% (27 - 35.5%) |  |
