# Supplementary material for: Impaired remyelination in late-onset multiple sclerosis
Source: Acta Neuropathol. 2025 Apr 1;149(1):30. doi: 10.1007/s00401-025-02868-5 (PMC11961469; doi:10.1007/s00401-025-02868-5)
Supplement: Supplementary file 5 — Supplementary file5 (DOCX 17 KB) [file 401_2025_2868_MOESM5_ESM.docx]

Supplementary Table 5: Correlation analyses of inflammatory cells, axonal density and acute axonal damage in non-demyelinated white matter and lesions with different demyelinating activities and non-MS controls with the **age of patients**

| Cell type (Marker) | **Non-demyelinated white matter**,  r-index and p-value  (Spearman correlation) | **Early active demyelinating lesions**  r-index and p-value  (Spearman correlation) | **Late active demyelinating lesions,**  r-index and p-value  (Spearman correlation) | **Inactive demyelinated lesions**,  r-index and p-value  (Spearman correlation) | **Healthy controls**  r-index and p-value  (Spearman correlation) |
| --- | --- | --- | --- | --- | --- |
| T cells (CD3) | r =0.3, p=0.07 | r = - 0.1, p=0.5 | r = - 0.04, p=0.8 | **r =0.7, p=0.04** | **-** |
| Cytotoxic T cells (CD8) | r =0.4, p=0.06 | r = - 0.2, p=0.17 | r =0.01, p=0.9 | **r =0.8, p=0.01** | **-** |
| B cells (CD20) | r =0.2, p=0.3 | r = - 0.2, p=0.3 | r = - 0.07, p=0.7 | r =0.7, p=0.07 | - |
| Plasma cells (CD138) | r = - 0.1, p=0.6 | r = - 0.2, p=0.2 | r = 0.1, p=0.5 | r = 0.5, p=0.3 | - |
| Microglia (KiM1P) | r = - 0.08, p=0.6 | r = - 0.02, p=0.9 | r = 0.03, p=0.8 | r = 0.1, p=0.7 | - |
| Macrophages (KiM1P) | r = 0.1, p=0.6 | r = - 0.2, p=0.1 | r = 0.09, p=0.6 | **r = 0.8, p=0.04** | **-** |
| Early activated macrophages (MRP14) | r = - 0.1, p=0.4 | r = - 0.2, p=0.1 | r = - 0.07, p=0.7 | r = 0.3, p=0.4 | - |
| Axonal spheroids / acute axonal damage (APP) | r = - 0.2, p=0.2 | **r=-0.3, p=0.02** | r = - 0.1, p=0.5 | r = - 0.09, p=0.8 | - |
| Myelinating oligodendrocytes (BCAS1) | **r = - 0.6, p= 0.004** | r = - 0.002, p=0.9 | r = - 0.04, p=0.8 | **r = - 0.6, p=0.01** | r = 0.3, p=0.2 |
| Oligodendrocyte precursor cells (strong Olig2) | r = - 0.02, p=0.9 | r = - 0.1, p=0.5 | r = 0.005, p=0.9 | r = 0.04, p=0.09 | r = - 0.2, p=0.4 |
| Mature oligodendrocytes (NogoA) | **r = - 0.5, p= 0.01** | r = - 0.2, p=0.3 | r = 0.007, p=0.9 | r = 0.15, p=0.5 | r = - 0.2, p=0.3 |
| Axonal density (Bielschowsky silver staining), % of axons relative to non-demyelinated white matter LOMS | | r = 0.09, p=0.6 | **r=0.3, p=0.04** | r = 0.7, p=0.13 | - |
