# Supplementary material for: Impaired remyelination in late-onset multiple sclerosis
Source: Acta Neuropathol. 2025 Apr 1;149(1):30. doi: 10.1007/s00401-025-02868-5 (PMC11961469; doi:10.1007/s00401-025-02868-5)
Supplement: Supplementary file 6 — Supplementary file6 (DOCX 12 KB) [file 401_2025_2868_MOESM6_ESM.docx]

Supplementary Table 6: Correlation of **BCAS1-positive cells** in non-demyelinated white matter and demyelinated lesions with **EDSS at last follow-up**

|  | **Non-demyelinated white matter**  r-index and p-value  (Spearman correlation) | **Early active demyelinating lesions**  r-index and p-value  (Spearman correlation) | **Late active demyelinating lesions**  r-index and p-value  (Spearman correlation) | **Inactive demyelinated lesions**  r-index and p-value  (Spearman correlation) |
| --- | --- | --- | --- | --- |
| **Correlation of BCAS1-positive cells and EDSS at last follow-up** | r = - 0.2, p=0.4 | r = - 0.2, p=0.3 | r = 0.2, p=0.6 | r = - 0.2, p=0.3 |
