# Supplementary figures and images for: Impaired remyelination in late-onset multiple sclerosis
Source: Acta Neuropathol. 2025 Apr 1;149(1):30. doi: 10.1007/s00401-025-02868-5 (PMC11961469; doi:10.1007/s00401-025-02868-5)

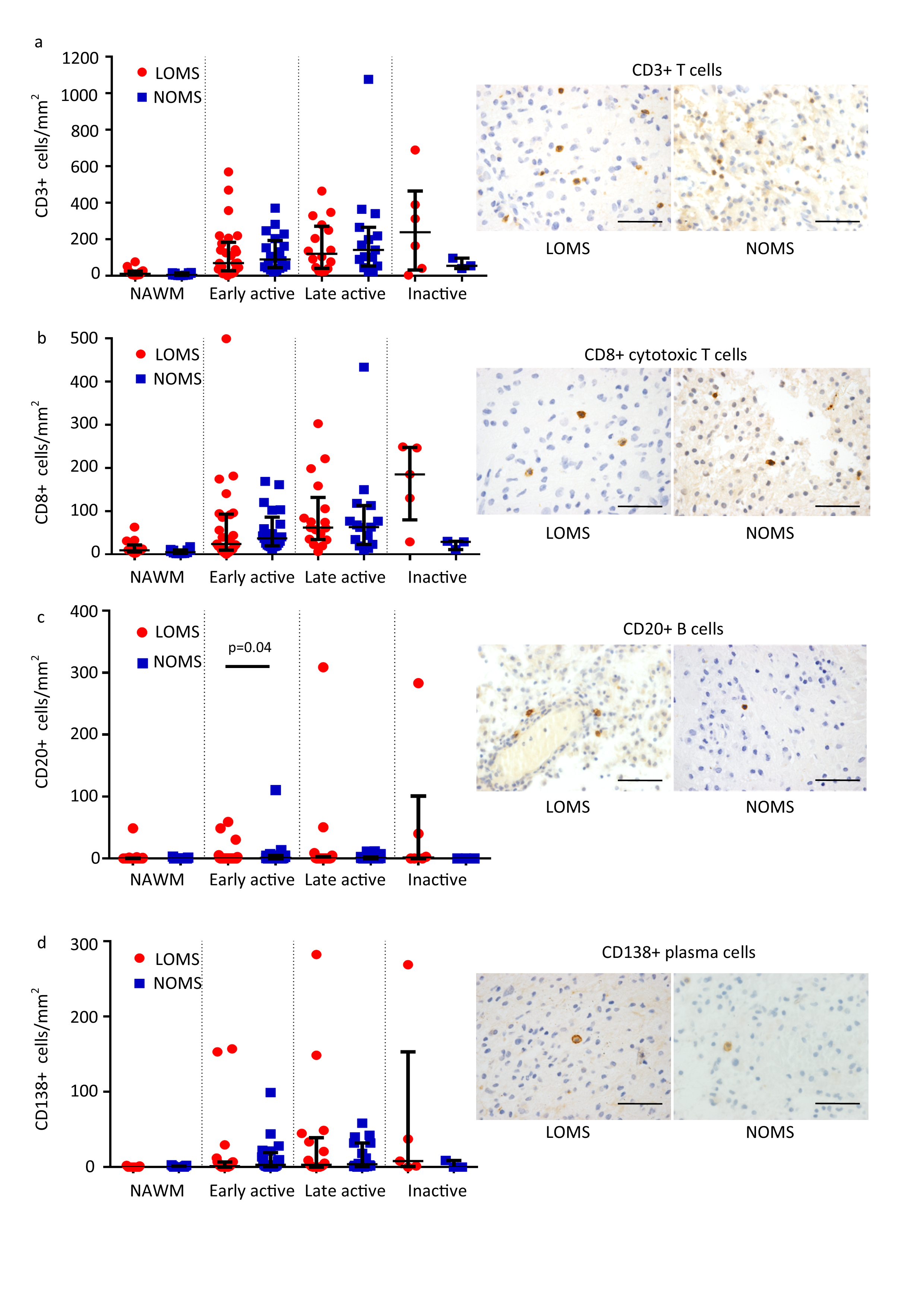

Supplement: Supplementary file 7 — Supplementary file7 (TIF 4088 KB) [file 401_2025_2868_MOESM7_ESM.tif]

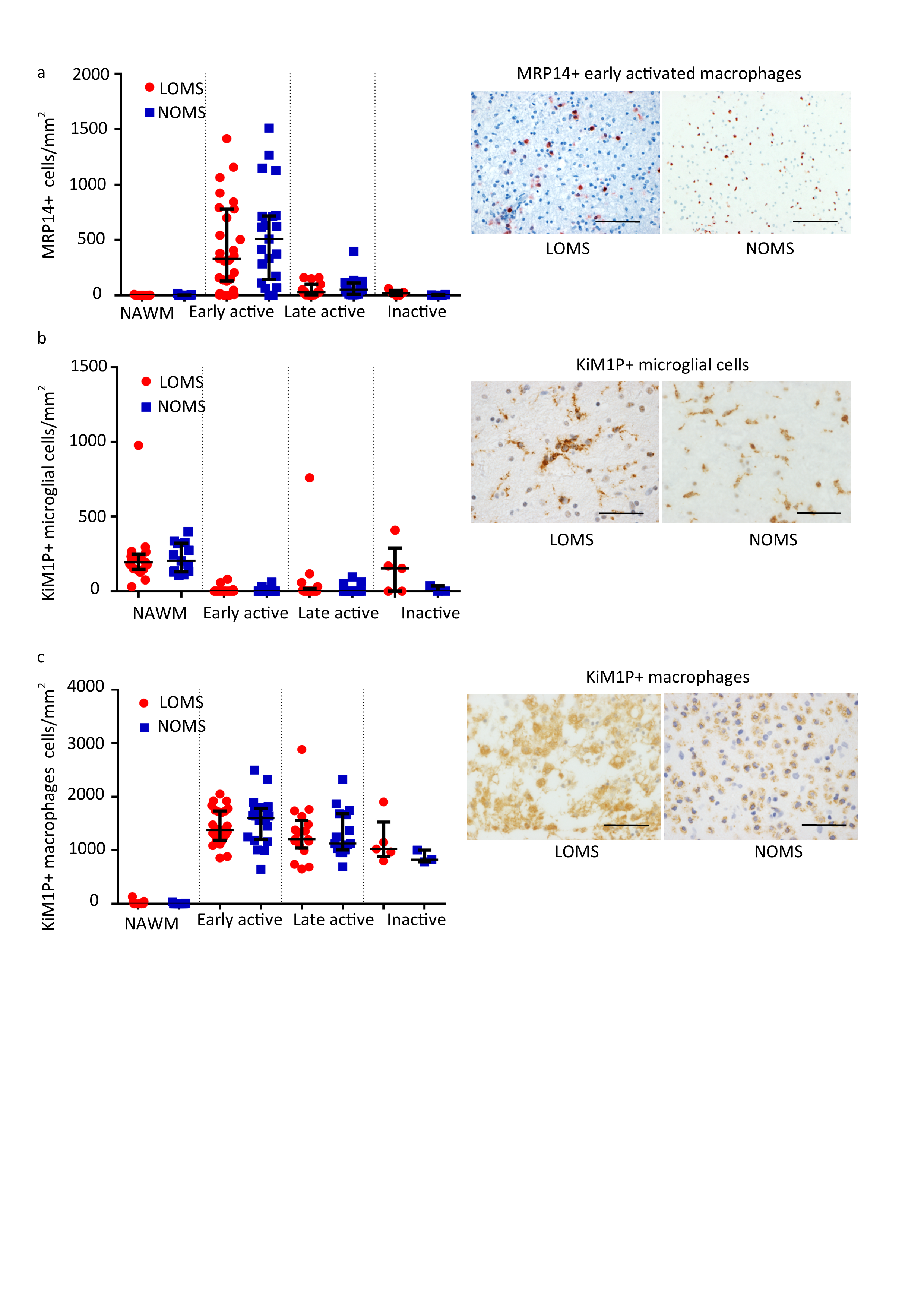

Supplement: Supplementary file 8 — Supplementary file8 (TIF 3252 KB) [file 401_2025_2868_MOESM8_ESM.tif]

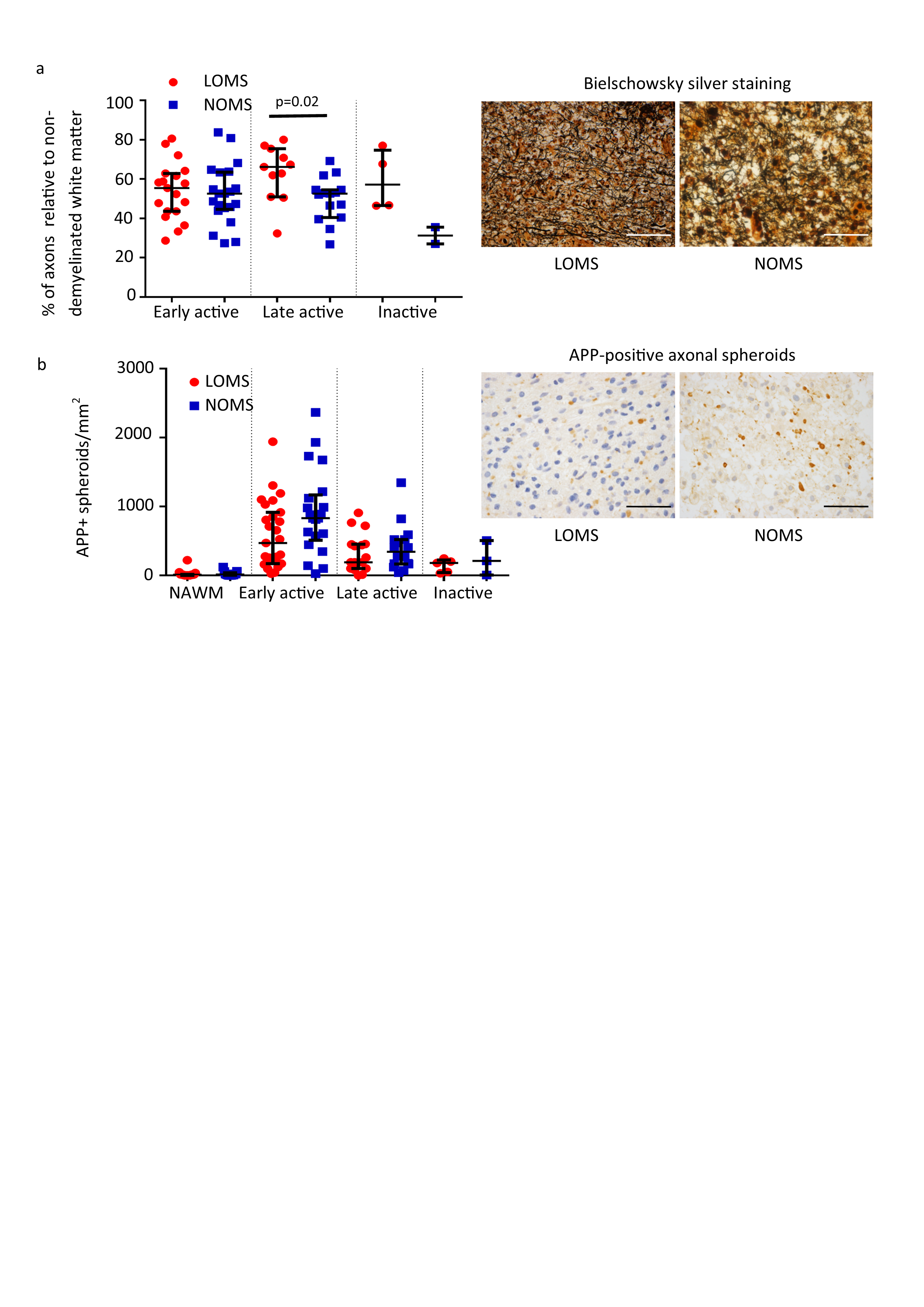

Supplement: Supplementary file 9 — Supplementary file9 (TIF 2991 KB) [file 401_2025_2868_MOESM9_ESM.tif]
